# ‘The Catastrophic World’: Capitalism, Climate Crisis, COVID-19, and C. Wright Mills

**DOI:** 10.1177/08969205221097308

**Published:** 2022-06-07

**Authors:** Zaheer Baber

**Affiliations:** University of Toronto, Canada

I have tried to be objective. I do not claim to be detached . . . you must not expect me to provide ‘A Balanced View’. I am not a sociological book-keeper.

C. Wright Mills

After trying his hand at ‘professional’ sociology for a research project directed by the relentless ‘professionalizer’ Paul Lazarsfeld, at Columbia University, C. Wright Mills eventually saw through the pointlessness of it all. Given the turbulent post-war heating up of the cold war in Southeast Asia, the politically passionate and engaged C. Wright Mills struggled and eventually refused to be contained and constrained by the vacuous, arid methodological and theoretical straightjackets that had resulted from the professionalization of Sociology project. In a 1951 letter to his editor at Knopf, Mills expressed deep dissatisfaction with the directions in which sociology was being pushed by its self-appointed gatekeepers. Without actually naming Paul Lazarsfeld and Talcott Parsons whose work he later trenchantly criticized, Mills wrote, sociology ‘is now split into statistical stuff and heavy-duty theoretical bullshit. In both cases, there’s no writing but only turgid polysyllabic slabs of stuff’ (Mills and Mills, 2000: 154–155).

A few years later, he famously articulated his misgivings on the post-war professionalizing project that threatened to tame and disembowel the critical legacies of the classical tradition in the now classic *The Sociological Imagination* (Mills, 2000a [1959]). His strident but by no means unfair critique of professionalization that for him threatened to decouple ‘scientific’ sociology from making sense of the vicissitudes of actual societies are too well known to recapitulate here (Aronowitz, 2012; Geary, 2009; Hayden, 2006; Summers, 2006, 2007, 2008). His goal in *The Sociological Imagination* was not simply to offer a critique of the rigid, stultifying paths that were then being paved for the putatively ‘professional’ and ‘scientific’ sociology by its gatekeepers. Instead, Mills who rode a motorcycle to Columbia University that he himself had assembled from parts he had received in crates, had built not one but two houses and had even tried his hand at growing his own food in his suburban New York backyard, also came up with alternative routes and possibilities for preserving and augmenting the valuable critical legacy of the classical sociologists.

As he formulated it, ‘the sociological imagination’ consisted of the seamless connection of history, biography, and social structure in an attempt ‘to translate personal troubles into public issues, and public issues into the terms of their human meaning for a variety of individuals’ (Mills, 2000a [1959]: 187). In contrast to those he labeled as ‘mere technicians’, the ‘crackpot realists’ or the ‘grand theorists’, Mills’ sought to construct ‘big picture’ accounts, narratives, and explanations of social struggles and contestations that simultaneously constitute the present and fuel possible social change. As he put it, is a sociologist’s

. . . responsibility to orient modern publics to the catastrophic world in which they live . . . but he (sic!) cannot do it if he remains a mere specialist. To do it all, he’s got to do it big . . . What they need, and what they feel they need, is a quality of mind that will help them to use information and to develop reason in order to achieve lucid summations of what is going on in the world and of what may be happening within themselves . . . They yearn for facts, they search for their meanings, they want ‘a big picture’ in which they can believe and within which they can come to understand themselves. The sociological imagination enables its possessor to understand the larger historical scene in terms of its meaning for the inner life and the external career of a variety of individuals . . . how individuals, in the welter of their daily experience, often become falsely conscious of their social positions. Within that welter, the framework of modern society is sought, and within that framework the psychologies of a variety of men and women are formulated. By such means the personal uneasiness of individuals is focused upon explicit troubles and the indifference of publics is transformed into involvement with public issues. (Mills and Mills, 2000:vii; Mills, 2000b [1959]: 5)

Emphatically rejecting the misinterpretation of Max Weber’s concept of ‘value neutrality’ and the ideological advocacy of allegedly non-ideological sociology, Mills famously asserted, ‘I have tried to be objective. I do not claim to be detached’ (Gouldner, 1970; Summers, 2008: 6).

Given the multiple challenges posed by Mills, the reactions from the professionalizing gatekeepers to the publication of The Sociological Imagination were unsurprisingly scathing, sneering, and dismissive. Some presented Mills as an uncouth non-sociological interloper who was trespassing on their intellectual private property. Thus, Edward Shils (1960) drew on the available regional stereotypes to mock Mills’ Texan background to ask,

what does this solitary horseman—who is in part a prophet, in part a scholar, and in part a rough-tongued brawler—a sort of Joe McCarthy of sociology, full of wild accusations and gross inaccuracies, bullying manners, harsh words, and shifting grounds want of sociology? (pp. 77–78)

In a follow-up piece, he warned fellow gatekeepers that ‘Professor Mills will continue to play his rat-catcher’s pipe; and the liveliest and most indomitable of the post-graduate students and young lecturers, who hold the future of the subject in their hands, will disappear into the hillside which, to them and to him, looks like a mountain’ (Shils, 1961: 621). Robert K. Merton—who with Paul Lazarsfeld, had not only personally hired him to work at Columbia but had previously considered him to be ‘one of the most talented American sociologists’, now labeled his The Sociological Imagination as the ‘little book by C. Wright Mills’ with its ‘violent attacks strafing sociology’ (Summers, 2006: 37). On his part, Paul Lazarsfeld who was one of the two main targets of Mills’ critique, exclaimed, ‘I find what Mills writes, you see, just ridiculous. There is nothing in the world he can, as a research man, contribute to anything . . . why doesn’t he leave us alone?’ (Summers, 2006: 37). Not to be outdone, Irving Louis Horowitz who, like Merton, had earlier called Mills ‘the greatest sociologist the United States has ever produced’, went on to dismiss him as ‘a prophet and a fanatic . . . bigot . . . a sadly flawed individual’ who ‘could no longer really be properly defined as being within the field of sociology’ (Summers, 2007: 116).

Clearly, in his attempt to remind the ‘professionalizers’ of the continuing relevance of the critical legacies of the classical sociologists, C. Wright Mills had more than just lightly stepped on some sensitive nerves. To be sure, not all the classical sociologists were critical thinkers. Following Comte’s positivism, many of the early American sociologists such as James Dealey, Edward Ross and Lester Ward among others were also heavily invested in ‘physics envy’ and ‘social physics’ (Calhoun, 2005; Turner, 2014). There were of course many other American sociologists such as WEB Du Bois (2015 [1903], 1996 [1899]; Burawoy, 2021a, 2021b; Morris, 2015, 2017), Thorstein Veblen (1899, 1918; Appiah, 2021), and the Lynds (1939) who had provided viable critical alternatives to the emergent positivist, structural-functionalist orthodoxies of the period.

But despite the many possible critical alternatives, for a time it seemed as if the project of the ‘professionalizers’ of American sociology might actually succeed in the mass production of what Max Weber (1958) termed ‘specialists without spirit, sensualists without heart. . . those little cogs’ (p. 182). Indeed, that might have well been the case had the mass protests and concerted resistance associated with the Civil Rights Movement, the anti-Vietnam War movement, and the countercultural challenges to business as usual, not broken out when they did. In those turbulent and tumultuous times, many graduate students, some almost firmly embedded in the ‘professionalizing’ sociology assembly-line, rebelled (Sica and Turner, 2005). Before long, several organizations and movements—the Association for Humanist Sociology; The Sociology Liberation Movement (Flacks, 1988; Lee, 1988; Lee and Lee, 1972; Levine, 2004; Murray, 1988; Nicolaus, 1968; Stark, 1988) emerged. The journals associated with these organizations—*Social Problems*, *The Insurgent Sociologist*, re-named later as *Critical Sociology*—provided the much-needed oxygen as well as intellectual space for alternatives to the technocratic lack of vision pushed by the ‘professionalizers’. Thousands of students, more than ever curious about the grindingly unequal social world and conflicts, flocked to the sociology departments in search of tentative answers that sometimes generated even more intriguing questions.

There is no doubt that ‘the disobedient generation’ (Sica and Turner, 2005) made the most of the turbulent social context by successfully pushing not just the academy but society too toward hitherto less explored critical pathways and directions. After all, several major progressive policy changes—The Fair Labor Standards Act, the Environmental Protection Agency, the Occupational Safety and Health Administration, the Federal Mine Safety and Health Act as well as the Clean Air and the Clean Water Act, and so on—were possible largely due to the pressure of the multiple social movements on the republican administration of Richard Nixon (Baber, 2016). At the same time, however, it did not take very long for pushback from the ideologues and the beneficiaries of the spoils of the emergent neoliberal capitalism. The substantial presence and power of critical scholars notwithstanding, most American universities were not immune to the neoliberal projects that were underway with a vengeance (Burawoy, 2022; Calhoun, 2022). In eagerly accepting and vying for academic stardom, endowed professorships and citation metrics in all their fine-tuned incarnations—the h-index, the i-index, and no doubt more to come—the obsessive ranking of universities as the ultimate measures of scholarly value, most scholars were not just passive victims and bystanders. Many were, of course, and continue to be quite enthusiastic active agents—with some indulging in hyper-masculinist games of braying about the length of their i-indexes—in the construction of a fateful hybrid of bureaucratic, privatized structures in which they now find themselves entangled (Allmer, 2019; Burawoy, 2016, 2022; Burrows, 2012; Fasenfest, 2010; Holmwood, 2010).

Among many others, it was the influential writings and the ‘sociological imagination’ of C. Wright Mills who also coined the term ‘new left’ that nurtured and supported alternative critical perspectives within the discipline. His book *The Power Elite* (Mills, 2000a [1959]) provided both a compelling analysis of the post-war transformations and changes in the structure of American capitalism and its implications for the future. Rejecting the

view that makes the big economic man the real holder of power; the simple liberal view that makes the big Political man the chief of the power system . . . [and those] who would view the warlords as virtual dictators. (Mills, 2000a [1959]: 277)

Mills (2000a [1959]) drew on both Marx and Weber, to focus on the emergence of a power elite that, in his words, ‘has been shaped by the coincidence of interest between those who control the major means of production and those who control the newly enlarged means of violence’ (p. 276). He continued,

. . . the economy is at once a permanent-war economy and a private-corporation economy. American capitalism is now in considerable part a military capitalism, and the most important relation of the big corporation to the state rests on the coincidence of interests between military and corporate needs, as defined by warlords and corporate rich . . . this . . . further subordinates the role of the merely political men . . . one is tempted to speak of a political vacuum in which the corporate rich and the high warlord, in their coinciding interests, rule. (Mills, 2000a [1956]: 276)

Mills (2000a [1959]) pointed out that after 1939, ‘the focus of elite attention has been shifted from domestic problems . . . to international problems’ (p. 275). It was this shift, from the onset from the Second World War to the cold as well as the forever wars that led to the making and the consolidation of global—to use Mills’ words—‘military capitalism’ or the military-industrial complex made popular by Eisenhower who, when he was the President of Columbia University, had attended Mills’ sociology lectures. As Mills (2000a [1959]) argued, ‘it is the professional politician that has lost the most . . . not politicians, but corporate executives, sit with the military and plan the organization of war effort’ (p. 276). It is Mills’ concept of ‘military capitalism’ as the dominant element of the ‘power elite’ connected to his argument about the shift of elite attention from domestic to international issues that provides a critical understanding of the both the nature of current global capitalism and its braiding with the military and the forever wars that show absolutely no signs of winding down.

The military has been one of the largest and most consistent polluters of the planet—from the years of nuclear tests in Marshall Islands to the routine operations of war machines globally (Crawford, 2022; Scranton, 2018; Simon, 1997; Smith-Norris, 2016). Even without any help from the military, capitalism by itself was the major contributor to environmental destruction globally (Ghosh, 2017, 2021; Klein, 2015; Malm, 2018; Moore, 2015; Patel and Moore, 2017; Plys and Lemert, 2021; Foster, 2000). Conjoined with warfare, the capacity of ‘military capitalism’ to wreak environmental havoc and accelerate the climate crisis has multiplied dramatically. Thanks to the military-industrial complex that Mills identified, commodity fetishism and unrelenting consumption that always fueled modern capitalism are now, seamlessly fused and infused with `patriotic’ and `jingoistic’ ideologies of consumption of commodities churned out by the very same industries that produce armaments too. It is the tragic trajectories, textures, and consequences of the globalizing ‘military capitalism’ first pinpointed and analyzed by Mills that are the prime contributors to the destruction of the natural habitats that paved the way for the semi-global zoonotic diseases such as SARS in the past and now, the now—thanks to the global logistical structures in place to service global ‘military capitalism’—a fully globalized COVID-19 pandemic with possibly more in the wings. Although Mills obviously had nothing to say about either the climate crisis or COVID-19, it is his insightful ‘big picture’ critical ‘sociological imagination’ in general and more specifically, his analysis of the changing structure of global ‘military capitalism’—or the braiding and fusion of its unquenchable thirst for cheaper, compliant labor and markets together with ideological justifications for ongoing wars orchestrated and amplified by a compliant media—that is indispensable for understanding the causes of the climate crisis and COVID-19. To borrow his words once again (Mills and Mills, 2000:vii; Mills and Mills, 2000:5), given the ‘catastrophic world we live in’, Mills’ critical ‘sociological imagination’ that seeks to connect ‘personal troubles’ to the ‘public issues of the social structure’ to construct ‘lucid summations of what is going on in the world . . . a big picture’ continues to be more important than ever.
